# Newly Designed Cysteine-Based Self-Assembling Prodrugs for Sepsis Treatment

**DOI:** 10.3390/pharmaceutics15061775

**Published:** 2023-06-20

**Authors:** Yuta Koda, Yukio Nagasaki

**Affiliations:** 1 Department of Materials Science, Faculty of Pure and Applied Sciences, University of Tsukuba, Tennoudai 1-1-1, Tsukuba 305-8573, Japan; koda@ims.tsukuba.ac.jp; 2 School of Medical Sciences, School of Comprehensive Human Sciences, University of Tsukuba, Tennoudai 1-1-1, Tsukuba 305-8573, Japan; 3 Center for Research in Radiation, Isotope and Earth System Sciences (CRiES), University of Tsukuba, Tennoudai 1-1-1, Tsukuba 305-8573, Japan

**Keywords:** PEG-*block*-poly(cysteine), block copolymers, sepsis, oxidative stress, reactive oxygen species (ROS), nanoparticle antioxidants, self-assembly, polymer drugs, cysteine

## Abstract

Reactive oxygen species (ROS) are essential signaling molecules that maintain intracellular redox balance; however, the overproduction of ROS often causes dysfunction in redox homeostasis and induces serious diseases. Antioxidants are crucial candidates for reducing overproduced ROS; however, most antioxidants are less effective than anticipated. Therefore, we designed new polymer-based antioxidants based on the natural amino acid, cysteine (Cys). Amphiphilic block copolymers, composed of a hydrophilic poly(ethylene glycol) (PEG) segment and a hydrophobic poly(cysteine) (PCys) segment, were synthesized. In the PCys segment, the free thiol groups in the side chain were protected by thioester moiety. The obtained block copolymers formed self-assembling nanoparticles (Nano^Cys(Bu)^) in water, and the hydrodynamic diameter was 40–160 nm, as determined by dynamic light scattering (DLS) measurements. Nano^Cys(Bu)^ was stable from pH 2 to 8 under aqueous conditions, as confirmed by the hydrodynamic diameter of Nano^Cys(Bu)^. Finally, Nano^Cys(Bu)^ was applied to sepsis treatment to investigate the potential of Nano^Cys(Bu)^. Nano^Cys(Bu)^ was supplied to BALB/cA mice by free drinking for two days, and lipopolysaccharide (LPS) was intraperitoneally injected into the mice to prepare a sepsis shock model (LPS = 5 mg per kg body weight (BW)). Compared with the Cys and no-treatment groups, Nano^Cys(Bu)^ prolonged the half-life by five to six hours. Nano^Cys(Bu)^, designed in this study, shows promise as a candidate for enhancing antioxidative efficacy and mitigating the adverse effect of cysteine.

## 1. Introduction

Reactive oxygen species (ROS), such as superoxide, hydroxyl radicals, and hydrogen peroxide, are powerful oxidants produced in the body to maintain the homeostasis of biological systems [1]. For example, mitochondrial ROS are involved in the electron transport chain for obtaining biological energy. Intracellular ROS are used as signaling molecules to maintain redox balance. In general, glutathione (GSH) and endogenous antioxidant proteins (e.g., catalase, superoxide dismutase, and glutathione peroxidase) are used to control the intracellular redox balance [2,3,4,5,6]. When large amounts of ROS are produced, the endogenous antioxidant system cannot scavenge excessively generated ROS, and the intracellular redox balance cannot be maintained. An excess amount of ROS which cannot be appropriately reduced by the endogenous antioxidant system is harmful because these species oxidize intracellular components such as DNA, proteins, and lipids [7]. The accumulation of this oxidative damage is called oxidative stress. In the case of cancer, for example, many immune cells such as macrophages invade tumor tissues and produce a large number of ROS, which act as bullets to combat uncontrollable cancer cells. However, it has also been reported that ROS activate several important signals, such as nuclear factor kappa B (NF-κB), which conversely activates the progression and metastasis of tumors [8,9,10,11,12]. Therefore, the generation of ROS in the tumor environment is a strategy for directly killing cancer cells, whereas scavenging ROS in the tumor environment is also a strategy for anticancer therapy. Many antioxidants have been developed to scavenge overproduced ROS. However, none of these effectively function as anticancer drugs. For example, it was previously reported that *N*-acetyl cysteine (NAC) inhibited cell growth in cancer [8,13,14]. However, a recently published meta-analysis did not confirm the efficacy of antioxidants, including NAC, for cancer treatment [15,16,17]. This is because small-molecule drugs diffuse into the entire body immediately after administration and are rapidly metabolized. Therefore, large or frequent doses are required to maintain efficacy; however, this causes severe adverse effects because small-molecule antioxidants are internalized in normal cells, causing dysfunction of the intracellular redox homeostasis. Thus, the conventional antioxidants developed to date have a narrow or no therapeutic window and show almost no effect for anticancer therapy.

Although the physical encapsulation of small-molecule drugs, including antioxidants, into nanoparticles such as liposomes and polymer micelles has been developed to reduce their adverse effects, the therapeutic efficacy is poor because the small-molecule drugs leak from the nanoparticles during circulation throughout the whole body [18,19,20,21]. Therefore, to reduce the immediate diffusion and metabolism of drugs, we are developing self-assembling nanoparticle drugs composed of hydrophilic poly(ethylene glycol) (PEG) and hydrophobic drug-based polymers bearing antioxidants and amino acids with covalent bonds [22,23,24,25,26]. In particular, amino acid-based self-assembling drugs release their corresponding amino acids through biodegradation by endogenous enzymes, and their therapeutic effects are enhanced by the release of amino acids [23,24,25,26]. For example, we previously designed poly(cysteine)-based self-assembling nanoparticle drugs (Nano^Cys(SS)^) whose thiol groups were protected by tertiary butylthio (S*t*Bu) groups and showed antitumor effects in a xenograft mouse model [26]. The results indicated that the *tert*-butyldithiol group was effectively cleaved in the tumor environment and induced an antioxidant effect there. Under the pathological conditions of acute inflammatory diseases, combined with cytokine storms, such as sepsis, ROS levels have been reported to rapidly increase simultaneously [27,28,29,30,31]. Therefore, the reactivity of disulfide bonds may not be sufficient for a rapid response to the treatment of such acute inflammatory diseases because ROS levels immediately increase with the overproduction of inflammatory cytokines throughout the body.

**Scheme 1:**
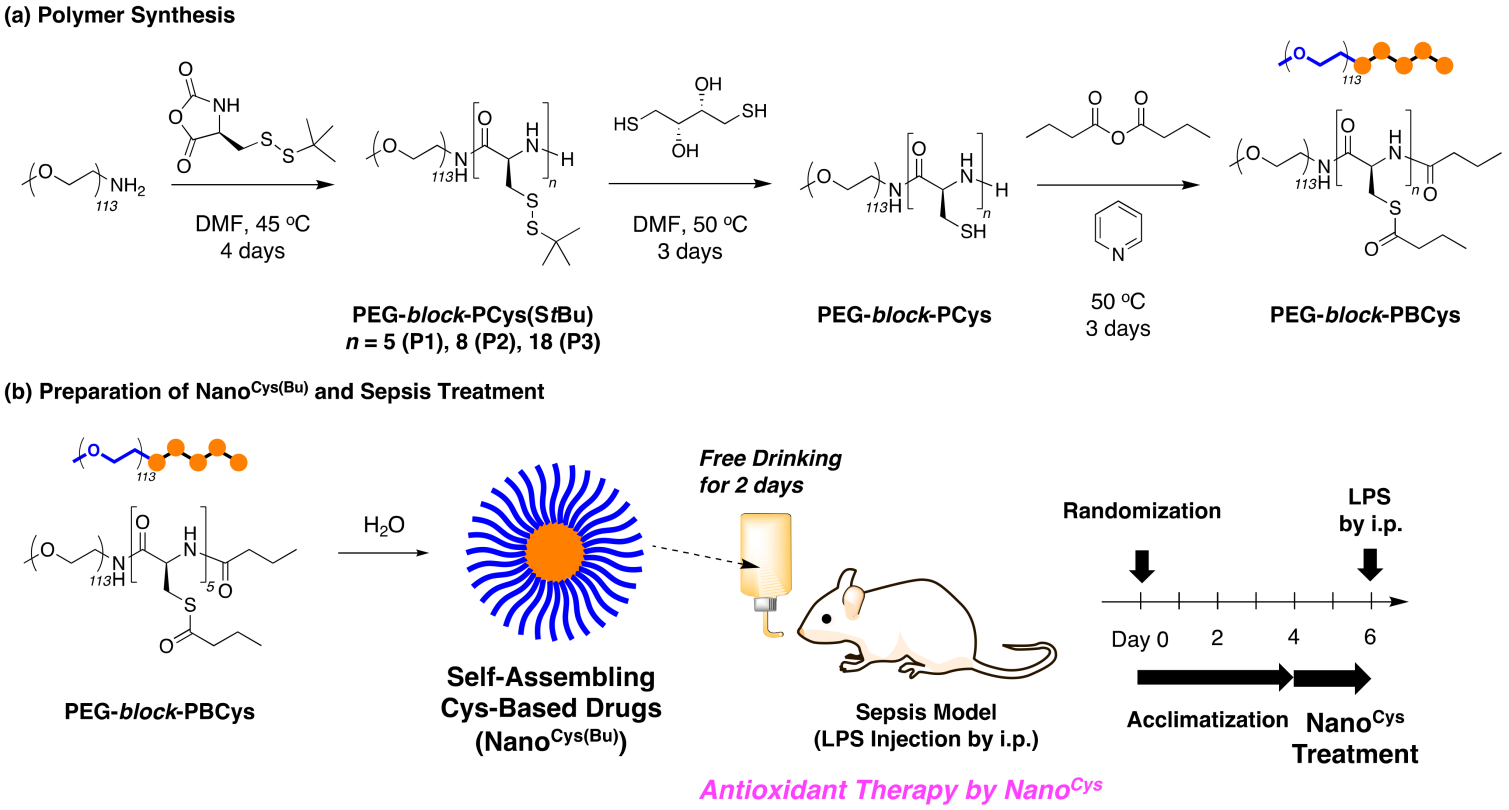
(**a**) Design of cysteine-based block copolymers by ring-opening polymerization of NCA-Cys(S*t*Bu), deprotection of disulfide bonds on PCys segments, and protection of free thiol groups with butyric anhydride. Polymerization condition: [NCA-Cys(S*t*Bu)]/[PEG-NH_2_] = 250/42.5 mM in DMF at 45 °C. Deprotection condition: disulfide bonds/_DL_-dithiothreitol (DTT) = 1/4 (mol/mol) in DMF at 50 °C. Protection reaction was caused by the excess amount of butyric anhydride in pyridine at 50 °C. (**b**) Protection of sepsis mice was induced by intraperitoneal injection of LPS by Nano^Cys(Bu)^ treatment. Nano^Cys(Bu)^(**P1**) was supplied by free drinking of the solution for two days before the LPS injection ([Cys] = 4.4 mM; [polymer] = 5.0 mg/mL in a drinking solution).

To this end, we designed new PEG- and poly(cysteine) (PCys)-based block copolymers whose thiol groups are protected by thioester bonds to enhance the reactivity of Nano^Cys^ against ROS, since thioester bonds exhibit high hydrolytic reactivity. These block copolymers were amphiphilic in order to form the nanoparticles with several nanometers in the aqueous condition. The nanoparticle structures enable us to avoid the cellular uptake by normal cells, preventing the disruption of the intracellular redox homeostasis. In addition, the extended bioavailability would suppress the oxidative damage caused by the severe systemic inflammation. To synthesize the targeted polymers, PEG-based block copolymers were synthesized through ring-opening polymerization of the *α*-amino acid *N*-carboxyanhydride of *S*-*tert*-butylmercapto-_L_-Cys (NCA-Cys(S*t*Bu)), with a PEG macroinitiator carrying an amino group end (Scheme 1a). The disulfide bonds were deprotected using a reducing agent, and the free thiol groups were protected using butyric anhydride to produce a new Cys-based block copolymer. The block copolymers formed the new nanoparticles (Nano^Cys(Bu)^) in water, and Nano^Cys(Bu)^ was stable under a variety of pH conditions. The obtained Nano^Cys(Bu)^ was applied to sepsis mice to investigate the potential of Nano^Cys(Bu)^. Nano^Cys(Bu)^ was supplied to mice by free drinking and was confirmed to prolong the half-survival life of the sepsis mouse models prepared by the intraperitoneal injection of lipopolysaccharides (LPS) compared to the non-treatment and cysteine administration groups (Scheme 1b). In this study, we have confirmed the enhanced pharmacokinetic characteristics of our nanoparticle-type antioxidants based on poly(cysteine). These antioxidants effectively suppress the adverse effects of cysteine, leading to improved therapeutic outcomes in sepsis treatments.

## 2. Materials and Methods

### 2.1. Materials

*S*-*tert*-butylmercapto-_L_-cysteine (Cys(S*t*Bu); Sigma-Aldrich, Saint Louis, MO, USA), triphosgene (TCI, purity > 98.0%), (1S)-(-)-α-pinene (TCI, purity > 97.0%), PEG-OH (Sigma-Aldrich, Saint Louis, MO, USA, *M*_n_~5000), methanesulfonyl chloride (MsCl; TCI, purity > 99.0%), triethylamine (Et_3_N; TCI, purity > 99.0%), chloroform (Wako, Tokyo, Japan, purity~99.0%), isopropanol (IPA; Wako, purity~98%), hexane (Wako, purity~95%), ammonia solution (Wako, ~28 wt%), 1,2,3,4-tetrahydronaphtalene (tetralin; TCI, purity > 98.0%), _DL_-dithiothreitol (DTT; TCI, purity > 98.0%), pyridine (Wako, purity~99.0%), butyric anhydride (TCI, purity > 98.0%), and trifluoroacetic acid (TFA; TCI, purity~98.0%) were used as received. Super-dehydrated tetrahydrofuran (THF; Kanto Chemicals, Tokyo, Japan, purity > 99.5%) and super-dehydrated *N*,*N*-dimethylformamide (DMF; Kanto Chemicals, purity > 99.5%) were further purified using a purification column (solvent dispensing system; glass contour; HANSEN & CO., Ltd., London, UK). Water for supplying the solution to the mice was prepared through the process of distillation and deionization using Milli-Q reference (Merck, Rahway, NJ, USA). Lipopolysaccharide (LPS from *Escherichia coli* O111:B4; Aldrich) was used as received.

### 2.2. Characterization

The number-averaged and weight-averaged molecular weights (*M*_n_, and *M*_w_, respectively) and the molecular weight distribution (*Đ*) of the polymers were measured using gel permeation chromatography (GPC) in DMF at 40 °C (flow rate: 0.40 mL/min) on two polystyrene gel columns (TOSHO TSKgel GMH_HR_-M; exclusion limit: molecular weight (MW) = 4.0 × 10^6^; particle size: 5 μm; pore size: N/A; 7.8 cm i.d. × 30 cm) connected to a pump (JASCO, PU-4180), a refractive index (RI) detector (JASCO, RI-2031), and a UV/Vis detector (JASCO, UV-4075). The columns were calibrated using 18 standard poly(ethylene oxide) (PEO) and poly(ethylene glycol) (PEG) samples (Merck; M_p_ = 238–1,180,000). The ^1^H nuclear magnetic resonance (NMR) spectra were recorded in CDCl_3_ or DMSO-*d*_6_ at room temperature (22–23 °C) using an AVANCE-600 NMR spectrometer (Bruker) operating at 600 MHz (^1^H). Dynamic light scattering (DLS) measurements were performed using a Zetasizer Nano ZSP (Malvern) equipped with a He–Ne laser (*λ* = 633 nm) at 37 °C ([polymer] = 1.0 mg/mL). The measurement angle was 173°, and the data were analyzed using the non-negative least squares (NNLS) method. Transmission electron microscopy (TEM) was performed using a JEM-1400 (JEOL) instrument at an accelerating voltage of 80 kV. The samples were prepared by drop-casting an aqueous solution of the micelles (10 mg/mL) onto carbon-coated grids (OKENSHOJI; ELS-C10 STEM Cu100P) and staining with a 1 wt% phosphotungstic acid solution (10 μL, pH = 7.4).

### 2.3. Synthesis of Cys-Based Block Copolymers

*α*-Amino acid *N*-carboxyanhydride of *S*-*tert*-butylmercapto-_L_-Cys (NCA-Cys(S*t*Bu)), a PEG macroinitiator carrying an amino end group (PEG-NH_2_), and PEG-*block*-PCys(StBu) were prepared using the method reported previously [26]. To prepare PEG-*block*-PCys(StBu) (**P1**(SS)), for example, PEG-NH_2_ (44.1 g, 8.82 mmol) was placed into a 1000 mL round-bottom flask, equipped with a three-way stopcock, and purged by N_2_ after drying under the reduced pressure. DMF (111 mL) and tetralin (0.5 mL) were added by a syringe technique under an N_2_ atmosphere. After the complete solubilization of PEG-NH_2_, the solution of NCA-Cys(StBu) in DMF (1000 mM, 52 mL, 52 mmol) was added to the flask, and the mixture solution was kept at 45 °C for 4 d. The polymerization was quenched by liq. N_2_ and purified by dialysis of the solution against methanol/water (=9/1, *v*/*v*) in a regenerated cellulose membrane (Spectra/Por 3^®^; molecular weight cut-off (MWCO) 1000).

#### 2.3.1. Deprotection of S-tert-Butylmercapto (StBu) Groups on PEG-*block*-PCys(StBu)

PEG-*block*-PCys(S*t*Bu) (**P1**(SS)) (90.1 g, 44 mmol of SS bonds; *M*_n_(GPC) = 6600, *Đ*(GPC) = 1.24, *DP*(Cys)_NMR_ = 5, *M*_n_(NMR) = 6000) was obtained by evaporation after dialysis against methanol (Spectra/Por^®^ 3; molecular weight cut-off (MWCO) = 1000). DMF (300 mL) was added, and the polymer was completely solubilized. DTT (27.2 g, 176 mmol; DTT/SS bonds = 4/1 mol/mol) was added, and the solution was purged with an N_2_ flow for 15 min. The solution was stirred at 50 °C for 3 d. The solution was then dialyzed against methanol (MWCO = 1000). The inner solution was evaporated, and PEG-*block*-PCys (**P1**(SH)) was obtained. GPC (DMF, PEO standard): *M*_n_(GPC) = 8200, *Đ*(GPC) = 1.74. ^1^H NMR (600 MHz, DMSO, r.t., *δ* = 2.50 ppm (DMSO)): δ 8.43–7.94 (brs, –COCH(CH_2_SH)N*H*–), 4.56–4.27 (brs, –COC*H*(CH_2_SH)NH–), 3.59–3.44 (brs, –OC*H*_2_C*H*_2_–), 3.23 (3H, s, –OC*H*_3_), 2.91–2.64 (brs, –C*H*_2_SH).

**P2**(SH) and **P3**(SH) with different molecular weights were synthesized in the same manner.

#### 2.3.2. Protection of Thiol Groups on PEG*-block*-PCys and Preparation of Micelle Solution

The obtained PEG-*block*-PCys (**P1**(SH)) was solubilized in pyridine (100 mL) and butyric anhydride (150 mL), and the solution was purged with N_2_ for 15 min. The solution was stirred at 50 °C for 3 d. The acylation reaction solution was directly dialyzed against water for preparing Nano^Cys(Bu)^(**P1**) (MWCO = 1000), followed by concentrating the obtained solution using a centrifugal evaporator (EYELA, CVE-3100). The concentration of the resulting solution was determined by weighing the obtained polymer after lyophilizing 100 μL of the solution. Finally, the solution was diluted with MilliQ water to achieve the target concentration. GPC (DMF, PEO standard): *M*_n_(GPC) = 7600, *Đ*(GPC) = 1.36. ^1^H NMR (600 MHz, CDCl_3_/TFA = 15/1 (*v*/*v*), r.t., *δ* = 7.26 ppm (CHCl_3_)): δ 8.32–7.45 (brs, –COCH(CH_2_SCO(CH_2_)_2_CH_3_)N*H*–), 4.89–4.60 (brs, –COC*H*(CH_2_SCO(CH_2_)_2_CH_3_)NH–), 3.96–3.58 (brs, –OC*H*_2_C*H*_2_–), 3.5 (3H, s, –OC*H*_3_), 3.44–3.10 (brs, –C*H*_2_ SCO(CH_2_)_2_CH_3_), 2.68–2.46 (brs, –SCOC*H*_2_CH_2_CH_3_), 1.80–1.53 (brs, –SCOCH_2_C*H*_2_CH_3_), 1.10–0.74 (brs, –SCOCH_2_CH_2_C*H*_3_).

Nano^Cys(Bu)^(**P2**) and Nano^Cys(Bu)^(**P3**) were prepared in the same manner, after the protection reactions of **P2**(SH) and **P3**(SH).

### 2.4. Animal Experiments

This study was conducted in strict accordance with the University of Tsukuba Guidelines for Animal Care and Laboratory Use, Japan (experimental plan approval #22-195). BALB/cA mice (seven weeks old, male) were purchased from Charles River Japan, Inc. (Yokohama, Japan). The mice were held under a light/dark cycle of 14 h/10 h at a temperature of 23.5 ± 2.5 °C and a humidity of 52.5% ± 12.5%.

#### 2.4.1. Preparation and Treatment of Sepsis Mouse Model

BALB/cA mice (body weight = 27–31 g) were randomized into four groups (N = 8) after seven days of acclimation. After randomization, the mice were further acclimatized to free drinking of Milli-Q water for four days. The aqueous solutions of Nano^Cys^ and Cys or MilliQ water were supplied to mice by free drinking for two days (Scheme 1b; [Cys] = 4.4 mM; [polymer] = 5.0 mg/mL). All mice received an intraperitoneal injection of LPS (LPS = 5.0 mg/kg-BW). The mice were then closely monitored for survival on an hourly basis.

#### 2.4.2. Statistical Analysis

Statistical analysis was conducted using GraphPad Prism 9 software (GraphPad Software Inc., Boston, MA, USA, 2020), and the survival curves were compared using the log-rank (Mantel–Cox) test.

## 3. Results

### 3.1. Polymer Synthesis

**Figure 1:**
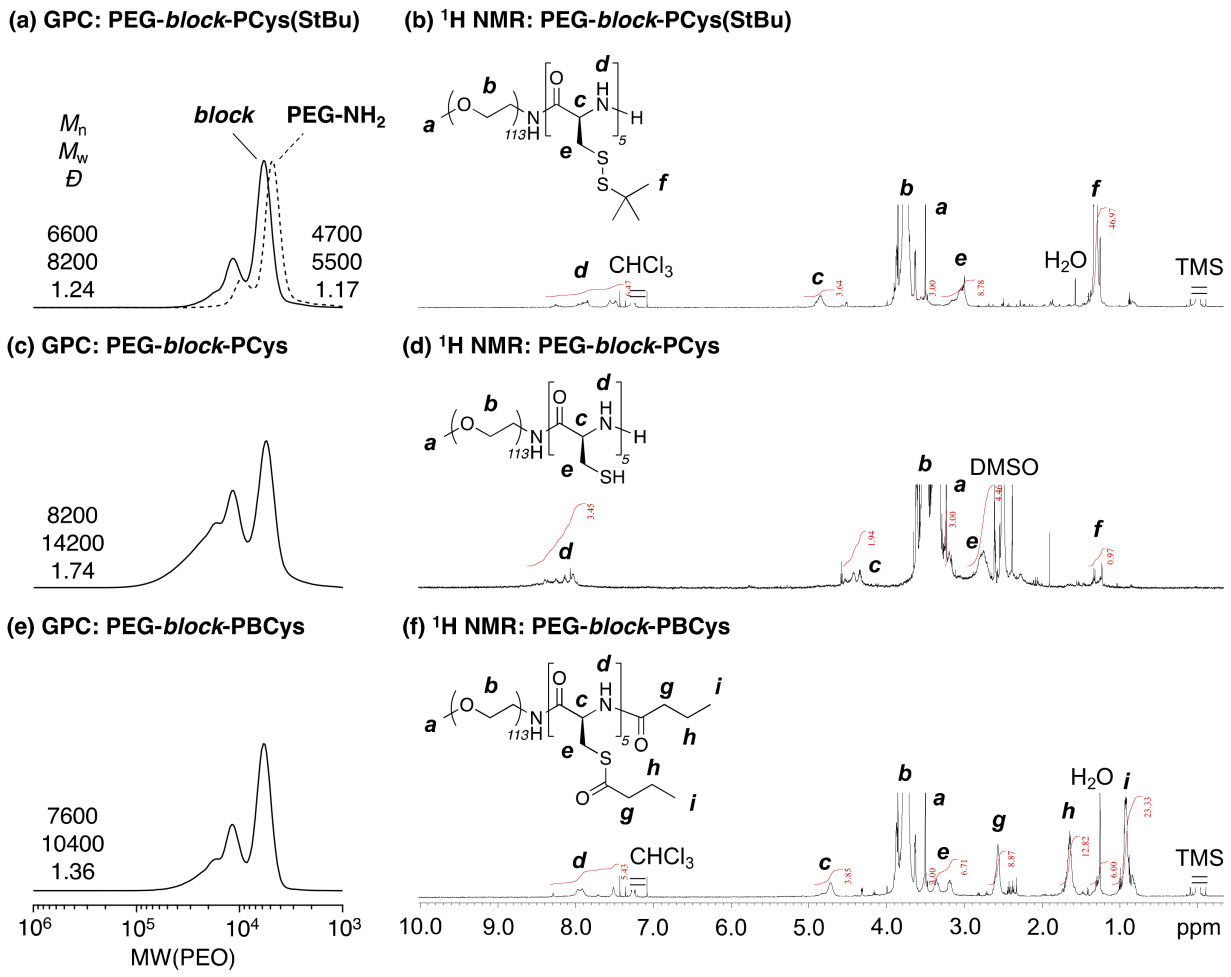
GPC curves and ^1^H NMR spectra (600 MHz; [polymer] = 5 mg/mL in (**b**,**f**) CDCl_3_/TFA = 15/1 (*v*/*v*) or (**d**) DMSO-*d*_6_) of cysteine-based block copolymers: (**a**,**b**), PEG-*block*-PCys(S*t*Bu) (**P1**(SS)); (**c**,**d**), PEG-*block*-PCys (**P1**(SH)); (**e**,**f**), PEG-*block*-PBCys (**P1**(Bu))).

A cysteine-based block copolymer was prepared in three steps: (1) the anionic ring-opening polymerization of the *α*-amino acid *N*-carboxyanhydride of *S*-*tert*-butyldithio-_L_-Cys (NCA-Cys(S*t*Bu)) with a PEG-NH_2_ macroinitiator, (2) the deprotection of the *tert*-butylmercapto groups by DTT, and (3) the protection of thiol groups by butyric anhydride in pyridine. First, NCA-Cys(S*t*Bu) was polymerized with PEG-NH_2_ in DMF at 45 °C, and 85% of the monomer was consumed in 96 h. With polymerization, the GPC curve shifted to higher molecular weights, suggesting that a block copolymer (PEG-*block*-PCys(S*t*Bu) **P1**(SS)) was obtained (Figure 1a, Table S1; *M*_n_(GPC) = 6600, *M*_w_(GPC) = 8200, *Đ*(GPC) = 1.24). All peaks in the ^1^H NMR spectrum were assigned to support the successful synthesis of PEG-*block*-PCys(S*t*Bu) (Figure 1b). From the areas of peaks ***a*** and ***f***, the degree of polymerization (DP) and absolute number-averaged molecular weight (*M*_n_(NMR)) were determined to be *DP*(Cys)_NMR_ = 5 and *M*_n_(NMR) = 5600, respectively. The *tert*-butyldithio groups on the PCys segments were cleaved by DTT in DMF at 50 °C for 3 d. The ^1^H NMR spectrum after deprotection showed that almost all of the peaks corresponding to the side chains disappeared, and the reaction yield was 98% (Figure 1d). Furthermore, the obtained thiol groups on the PCys segments were protected by butyric anhydride in pyridine at 50 °C for 3 d to obtain a PEG and PCys block copolymer carrying the butyroyl groups on the PCys segments (PEG-*block*-PBCys, **P1**(Bu)). Characterized by ^1^H NMR, the protection efficiency was almost 100%, and *M*_n_(NMR) = 5900 (Figure 1f).

**Figure 2:**
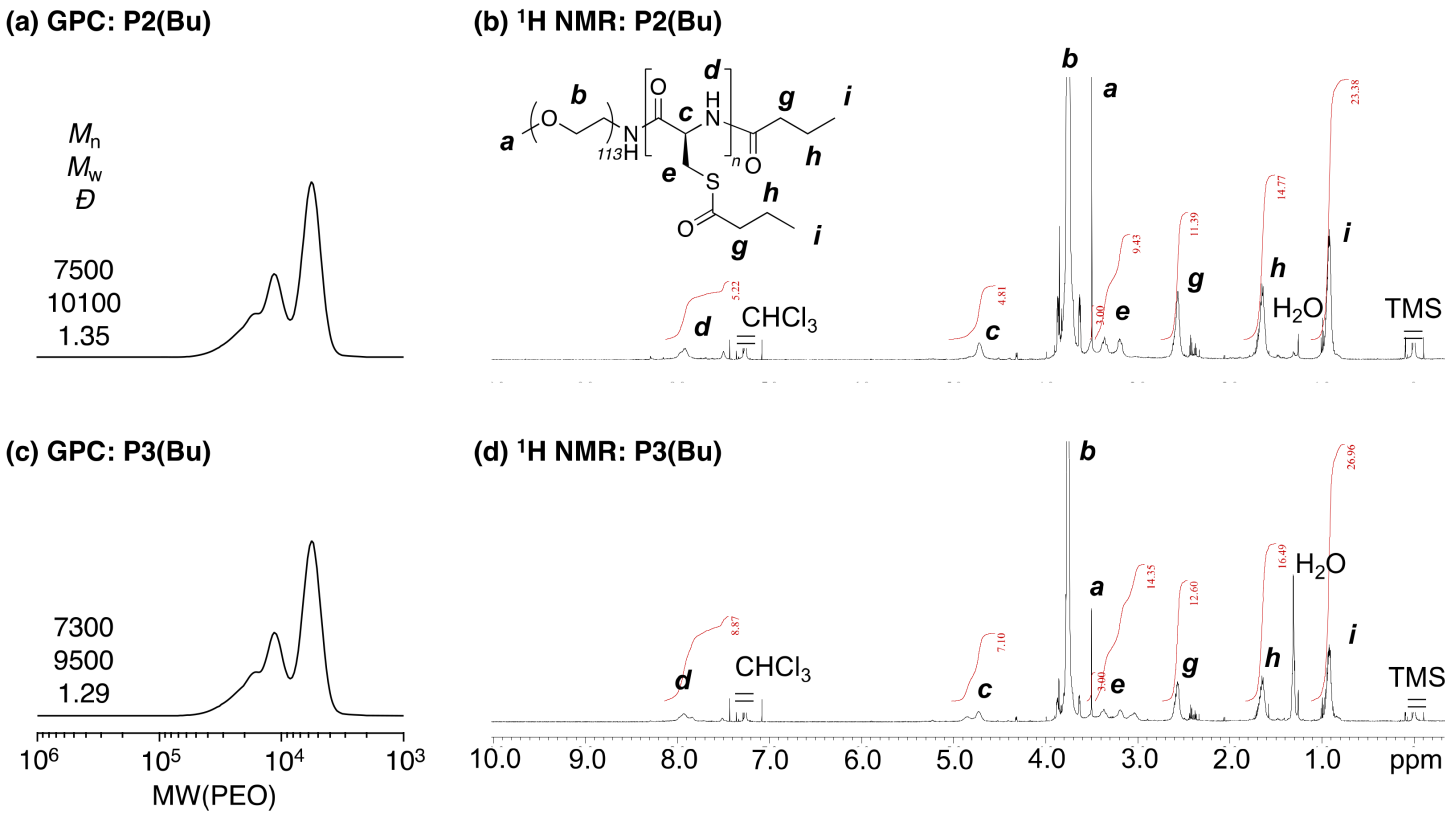
GPC curves and ^1^H NMR spectra (600 MHz; [polymer] = 5 mg/mL in CDCl_3_/TFA = 15/1 (*v*/*v*)) of PEG-*block*-PBCys: (**a**,**b**), **P2**(Bu); (**c**,**d**), **P3**(Bu)).

PEG-*block*-PBCys copolymers with different molecular weights were synthesized by changing the initial NCA monomer/PEG-NH_2_ ratio, and the target *DP*(Cys) values were 10 and 20. After the ring-opening polymerization, the successful synthesis of the copolymers was confirmed by GPC and ^1^H NMR (Figure S1, Table S1; *M*_n_(GPC) = 5900 (**P2**(SS)), 6900 (**P3**(SS)); *M*_w_(GPC) = 8000 (**P2**(SS)), 8400 (**P3**(SS)); *Đ*(GPC) = 1.21(**P2**(SS)), 1.15(**P3**(SS))). The *DP*(Cys)_NMR_ values were determined to be 8 and 18, which are close to the target values, and the *M*_n_(NMR) values were calculated as 6500 and 8400, respectively. Despite the different molecular weights, the deprotection reaction of the disulfide bonds on the PCys segments proceeded quantitatively with DTT, as confirmed by ^1^H NMR (Figure S2). Furthermore, the protection reaction of the free thiol groups on the PCys segments was also successful, and PEG-*block*-PBCys copolymers with different molecular weights were obtained (Figure 2, Table S1; *M*_n_(GPC) = 7500 (**P2**(Bu)), 7300 (**P3**(Bu)); *M*_w_(GPC) = 10,100 (**P2**(Bu)), 9500 (**P3**(Bu)); *Đ*(GPC) = 1.35 (**P2**(Bu)), 1.29 (**P3**(Bu))).

### 3.2. Characterization of Cysteine-Based Nanoparticles, Nano^Cys(Bu)^

**Figure 3:**
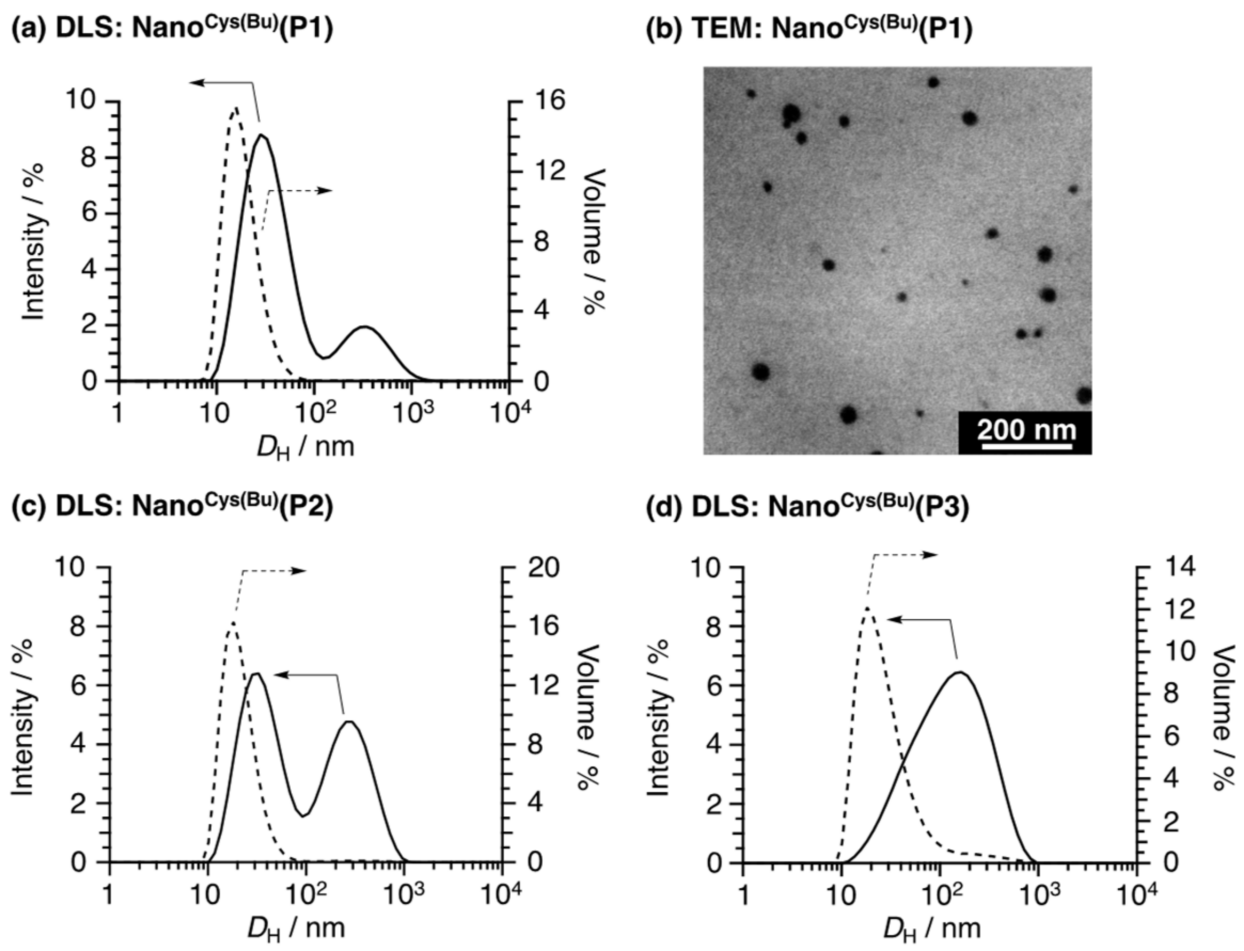
(**a**,**c**,**d**) Light scattering intensity (solid line) and volume (dash line) distribution of Nano^Cys(Bu)^ in water at 37 °C ([polymer] = 1.0 mg/mL), characterized by DLS. (**b**) TEM image of Nano^Cys(Bu)^(**P1**) cast on the carbon grid from the aqueous solution of Nano^Cys^ and stained by phosphotungstic acid.

Nanoparticles of PEG-*block*-PBCys (Nano^Cys(Bu)^) were prepared by direct dialysis of the reaction mixture against water, and Nano^Cys(Bu)^ was characterized using DLS and TEM measurements. The DLS measurement of Nano^Cys(Bu)^(**P1**) revealed two peaks based on the scattering intensity (*D*_H,intensity_; 37 and 376 nm), whereas no large peak was observed based on the volume distribution (*D*_H,volume_; 19 nm), indicating that almost no large aggregates were present (the intensity peak strength is proportional to 10^6^ times their size) (Figure 3a, Table S1). This is in good agreement with the TEM measurement results, in which Nano^Cys(Bu)^(**P1**) in the obtained image showed a size similar to that of the DLS data without large aggregates (Figure 3b). Nano^Cys(Bu)^(**P2**) and Nano^Cys(Bu)^(**P3**) were prepared in the same manner as Nano^Cys(Bu)^(**P1**), and these Nano^Cys(Bu)^ samples were also characterized by DLS and TEM (Figure 3c,d, Figure S3, Table S1). The resulting diameters, *D*_H,intensity_, were 37 nm (Nano^Cys(Bu)^(**P2**)) and 102 nm (Nano^Cys(Bu)^(**P3**)) in water, (Figure 3c,d).

**Figure 4:**
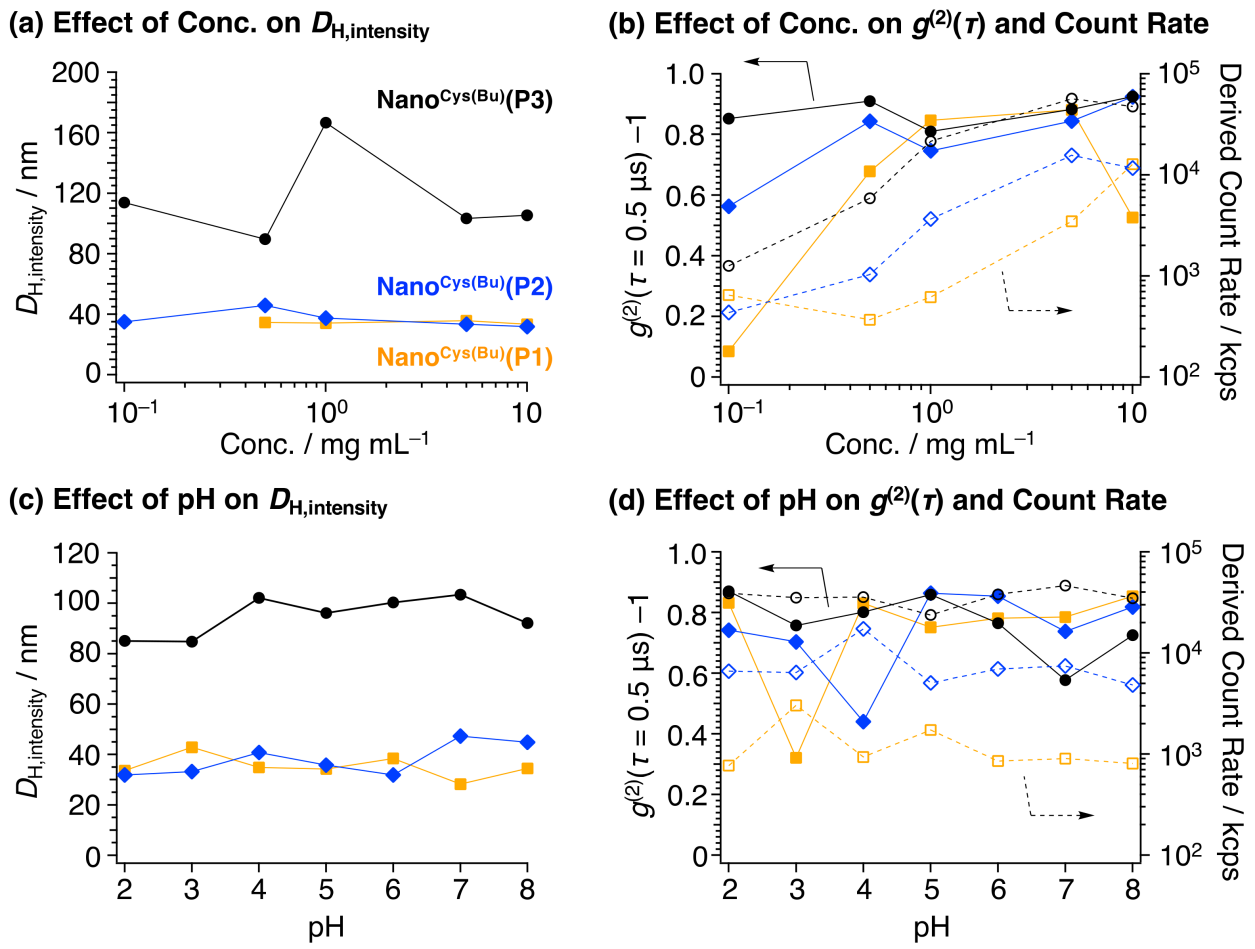
Effect of the polymer concentration (**a**,**b**) and pH (**c**,**d**) on the Nano^Cys^ formation in water at 37 °C (orange, Nano^Cys(Bu)^(**P1**); blue, Nano^Cys(Bu)^(**P2**); black, Nano^Cys(Bu)^(**P3**)). Effect of these factors on each *D*_H,intensity_ (**a**,**c**) and on the initial values of autocorrelation functions (*g*^(2)^(*τ* = 0.5 μs)–1; solid lines) and the derived count rate (dash lines) (**b**,**d**). The polymer concentration was set as 1.0 mg/mL (**c**,**d**).

We previously confirmed that, owing to their high stability and small size, core–shell-type nanoparticles strongly accumulate in the intestinal mucosa [32]. Therefore, the stability of Nano^Cys(Bu)^ was characterized by the changes in its concentration and pH (Figure 4, Figures S4 and S5). The concentration effect was analyzed in water from 0.1 to 10 mg/mL at 37 °C. In the case of Nano^Cys(Bu)^(**P1**), the initial value of the autocorrelation function (ACF; *g*^(2)^(*τ* = 0.5 μs)–1) under the concentration of 0.1 mg/mL was less than 0.5, while the corresponding values for the other two nanoparticles were greater than 0.5. These results indicate that Nano^Cys(Bu)^(**P1**) was not formed below 0.1 mg/mL. Under the other conditions, all of the nanoparticles maintained nearly the same size. The stability of Nano^Cys(Bu)^ under various pH conditions was investigated from pH 2 to 8 using a polymer concentration of 1.0 mg/mL. Regardless of the change in pH, the ACF and hydrodynamic diameters of all of the Nano^Cys(Bu)^ samples, as well as their distributions of light scattering intensity, did not change significantly (Figure 4c,d).

### 3.3. Application to Disease Model Mice

**Figure 5:**
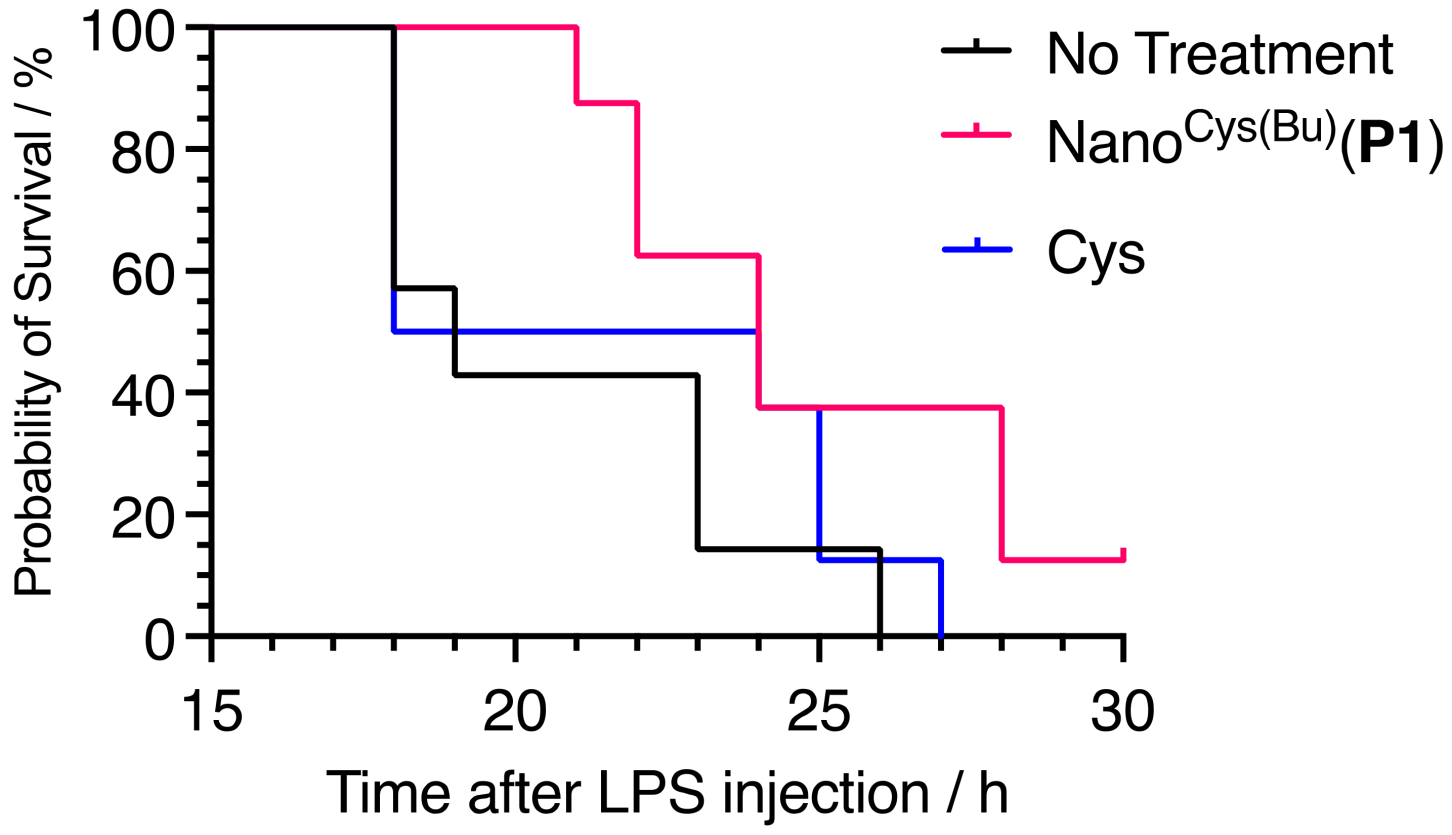
Nano^Cys(Bu)^ treatment (N = 8; LPS, 5.0 mg/kg-BW; [Cys] = 4.4 mM ([polymer] = 5.0 mg/mL) in a drinking solution) protection of mice from sepsis shock induced by intraperitoneal injection of LPS. The survival was expressed by Kaplan–Meier plots.

To investigate the potential of Nano^Cys(Bu)^ for sepsis treatment, sepsis model mice were prepared by LPS injection, and their survival time was analyzed. Nano^Cys(Bu)^(**P1**) was used for this study. After LPS was intraperitoneally injected (5 mg/kg-BW), the survival of the treated mice was monitored every hour. All of the sepsis model mice that did not undergo treatment died within 26 h, and similarly, the mice treated with Cys also died within 27 h (Figure 5). In contrast, the mice treated with Nano^Cys(Bu)^ survived until 30 h after the injection of LPS. Furthermore, the half–survival times in the no-treatment, Cys, and Nano^Cys^ groups were 19, 18, and 24 h, respectively. Nano^Cys^ thus prolonged the half-survival time by 5–6 h compared with the results for the no-treatment and Cys groups.

## 4. Discussion

Several diseases are associated with acute or chronic inflammation. In most cases, ROS are overproduced at the inflammatory sites. For example, after influenza infection in mice, the number of viruses increases rapidly, followed by an increase in the severity score and ROS level for several days up to one week [33,34,35]. Therefore, it is crucial to maintain the efficiency of antioxidant therapy for an extended period, without harmful effects. As explained in the Introduction, the problems associated with conventional antioxidants are their rapid diffusion and excretion, owing to their small size. In addition, small antioxidants induce dysfunction in the intracellular redox balance after internalization in normal cells. Therefore, conventional antioxidants cannot be used to treat most oxidative-stress-related diseases. Cysteine is a naturally occurring amino acid with a reduction potential. One of the well-known synthetic derivatives, *N*-acetylcysteine (NAC), has strong antioxidant properties, but it induces powerful adverse effects. To solve the problems of low molecular weight antioxidants, especially cysteine, we designed new Cys-based polymers which self-assemble in aqueous media to form core–shell-type nanoparticles covered with biocompatible PEG chains to develop antioxidant chemotherapy. Our idea was to extend the bioavailability of antioxidants by increasing the size of the self-assembled nanoparticles. It is also anticipated that a decrease in cellular uptake will prevent the dysfunction of intracellular redox homeostasis [36]. The disulfide protective group was converted to a thioester group to increase the deprotection speed, anticipating an increase in the antioxidant capacity. The thioester group is known to be much more active in hydrolysis than is the conventional ester group; therefore, we confirmed its self-assembling character, as well as its stability against increased pH and concentration. Nano^Cys(Bu)^, thus prepared, was applied for sepsis treatment due to its significant association with oxidative stress, its rapid deterioration in pathology, and its induction of systemic inflammation.

To prepare the PCys segment in the block copolymer, NCA-Cys(S*t*Bu) was directly polymerized with PEG-NH_2_. The alkyldithio group was used as a protecting group for the free thiol group in cysteine because acyl-protection is not sufficiently stable in NCA ring-opening polymerization [37]. Disulfide bonds on the PCys segments were cleaved by DTT in DMF at 50 °C after the polymerization of NCA-Cys(S*t*Bu), and PEG-*block*-PCys (**P1**(SH)), which possess a free thiol group as a side chain, was obtained. In this deprotection reaction, the disulfide bonds were almost completely cleaved because of the excess amount of DTT (disulfide bonds/DTT = 1/4 mol/mol), as confirmed by ^1^H NMR spectra before and after the reaction (Figure 1b,d). The GPC curve of PEG-*block*-PCys (**P1**(SH)) became broader than that before deprotection (PEG-*block*-PCys(StBu) (**P1**(SS)), probably because of the aggregation of the polymer chains (Figure 1a,c). The GPC curve of PEG-*block*-PBCys (**P1**(Bu)) after the protection reaction with butyric anhydride was narrower than that of PEG-*block*-PCys (**P1**(SH)) (Figure 1c,e). Therefore, the broadening of the GPC profile of **P1**(SH) was not due to the crosslinking of free thiol groups in the side chain of the polymer, but rather due to aggregation of the polymer through hydrogen bonding of the thiol groups.

The composition of the obtained block copolymers, PEG-*block*-PCys(S*t*Bu), could be easily controlled by the initial concentration ratio of [Cys-NCA] versus [PEG-NH_2_] and the three different prepared samples (**P1**(SS), **P2**(SS), **P3**(SS)); the degree of polymerization (DP) was confirmed by these ^1^H NMR spectra (Figure 1, Figure S1, Table S1; *DP*(Cys)_NMR_ = 5 (**P1**(SS)), 8 (**P2**(SS)), and 18(**P3**(SS))). PEG-*block*-PBCys copolymers (**P1**(Bu)–**P3**(Bu)) were successfully synthesized using butyric anhydride in pyridine. These molecular weights, determined by GPC, did not increase significantly (Figure 2a,c), probably because of the absorption of the polymers on the substrates of the GPC columns used. The longer PCys segments would interact with the GPC columns more than would the other copolymers. The ^1^H NMR spectrum of **P1**(Bu) suggested that the number of acyl-protecting groups in **P1**(Bu) was 6, determined by peaks ***g***, ***h***, and ***i*** (Figure 1f). Thus, one polymer chain of **P1**(Bu) had five acyl-protecting groups on the side chains and another on the terminal end chain. Therefore, all thiol and amino end groups were quantitatively modified, and the main chain of thet PCys segments was not affected through the deprotection and protection reactions at all. The ^1^H NMR spectra of **P2**(Bu) and **P3**(Bu) indicated that these polymers had 7 and 8 acyl-protecting groups, respectively (Figure 2b,d). The smaller than expected values suggested that these polymers showed much stronger molecular interactions than did **P1**(Bu), and these molecular dynamics decreased. According to the results using the **P1**(Bu)-based copolymers, **P2**(Bu) and **P3**(Bu) were also successfully synthesized, but the extent of the modification efficiency decreased due to the decreased molecular interaction.

Cysteine-based antioxidant polymer nanoparticles (Nano^Cys(Bu)^) were prepared by direct dialysis of the acylation reaction mixture against water. After the dialysis, Nano^Cys(Bu)^ was characterized by DLS and TEM. The hydrodynamic diameter (*D*_H,intensity_) of Nano^Cys(Bu)^(**P1**) according to scattering intensity showed the bimodal distributions, the size of which was 37 and 376 nm (Figure 3a, Table S1). Here, the large aggregates should be nearly negligible, as the light scattering intensity is emphasized because it is proportional to ten to the power of six of the size [38]. Actually, the volume-averaged hydrodynamic diameter, *D*_H,volume_, of Nano^Cys(Bu)^(**P1**) in water was 19 nm, and no large aggregate was observed. Therefore, the large nanoparticles scarcely existed in the solution. This was also confirmed by the TEM image, showing the particles with tens of nanometer (Figure 3b). Nano^Cys(Bu)^(**P2**) and Nano^Cys(Bu)^(**P3**) showed slightly larger sizes compared to Nano^Cys(Bu)^(**P1**) (Table S1; *D*_H,volume_ = 20 (**P1**), 26 (**P2**), 39 (**P3**) nm). However, this small difference is unlikely to have a significant effect on in vivo toxicity.

Based on these results, we have confirmed the preparation of cysteine-based nanoparticles with several tens of nanometers, possessing acyl-protective groups as a side chain. Before we applied the obtained Nano^Cys(Bu)^ to sepsis model mice, their stability was investigated in terms of pH and concentration. The effect of the polymer concentration on the nanoparticle formation was investigated by changing the concentration from 0.1 to 10 mg/mL. The initial value of the ACF (*g*^(2)^(*τ* = 0.5 μs)–1) at 0.5 μs is the important physical parameter for determining the micelle formation. When polymer micelles are formed, the initial value of the ACF is expected to be greater than 0.5 [38]. In the case of Nano^Cys(Bu)^(**P1**)), at a concentration of 0.1 mg/mL, the value was found to be less than 0.5, suggesting the absence of self-assembling nanoparticles, possibly due to the weaker coagulation force of the hydrophobic PCys segment (Figure 4b and Figure S4a). However, as the hydrophobic PCys segment increased, the ACF values exceeded 0.5, indicating the formation of a self-assembling structure. The size of the self-assembled structures of Nano^Cys(Bu)^ showed nearly the same hydrodynamic diameters (Figure 4a and Figure S4b). Since the pH in the digestive tract varies widely from 2 to 8, the effect of pH on the nanoparticle formation was also investigated. As a result, none of the Nano^Cys(Bu)^ samples significantly changed their size or ACF (Figure 4d and Figure S5), indicating that Nano^Cys(Bu)^ would be stable traveling from the stomach to the intestinal area, although the newly designed Nano^Cys(Bu)^s possess thioester groups in the side chain of the PBCys segment. Due to the fact that the amino-end group of the PCys chain was converted to an amide group by acylation, these polymers are not susceptible to pH variations. Further investigation on the physicochemical characteristics will be conducted, and the results will be published elsewhere.

Finally, the effect of the newly designed Nano^Cys(Bu)^ on a sepsis shock model in mice was investigated. Sepsis is a severe systemic inflammatory response syndrome (SIRS) that causes multiorgan dysfunction and blood clots, and the mortality rate is high, especially in the case of elderly and infant patients. The mechanism has not been completely elucidated; however, the cytokine storm caused by the overproduction of inflammatory cytokines, such as interleukin-6 (IL-6) and tumor necrosis factor-alpha (TNF-*α*), is considered as a key target [27,28,29,30,31]. Recently, inhibitors of these cytokines have been developed [30]; however, it remains difficult to immediately suppress the overproduction of cytokines. Furthermore, accompanied by the production of cytokines, a large amount of ROS is rapidly generated simultaneously, an event which is called a radical storm. The overproduction of ROS causes damage to cells and tissues throughout the body [27,28,33,34,35]. Thus, antioxidants have a great potential for application in the treatment of sepsis. However, low molecular weight antioxidants are ineffective against sepsis. Several methods can be used to induce sepsis in mice, including the intraperitoneal (i.p.) injection of lipopolysaccharides (LPS), cecal ligation and puncture, and the intravenous injection of bacteria. For the present study, we chose the i.p. LPS model due to its well-established capacity to induce significant ROS generation and the development of sepsis syndrome. In this study, Nano^Cys(Bu)^ and Cys were supplied to BALB/cA mice by free drinking for two days, and the sepsis shock model was induced by the intraperitoneal injection of LPS (LPS = 5.0 mg/kg-BW). We used Nano^Cys(Bu)^(**P1**) here, as it is stable at high concentrations, but tends to disintegrate at low concentrations. We anticipated that it would form stable spherical structures in the GI tract. However, it tends to disintegrate after migrating into the intestinal mucosa, which can be anticipated to increase the speed of enzymatic hydrolysis. In fact, we have previously confirmed a more pronounced impact of shorter PCys segments on inhibiting tumor growth [26]. Oral administration of Cys itself did not show any therapeutic effect; indeed, the half-survival time was almost the same as that in the no-treatment group. In contrast, Nano^Cys(Bu)^(**P1**) prolonged the half-survival time compared with that of the control group. Therefore, our self-assembling poly(cysteine)-based nanoparticles, Nano^Cys(Bu)^, have great potential for improving sepsis treatment. A detailed investigation into the effects of Nano^Cys(Bu)^ on sepsis treatment will be performed, and the results will be published in the future.

## 5. Conclusions

We designed and synthesized new Cys-based nanoparticles using PEG-based poly(cysteine) block copolymers. The block copolymers were prepared through ring-opening polymerization of NCA-Cys(S*t*Bu) and deprotection of the disulfide bonds on the PCys side chains, followed by protection of the thiol groups by butyric anhydride. These deprotection and protection reactions proceeded efficiently, without any main-chain scission reactions. Furthermore, the molecular weight of the PCys segments could be varied by modifying the ratio of [Cys-NCA] to [PEG-NH_2_]. The nanoparticles, Nano^Cys(Bu)^, were easily prepared by dialyzing the protection reaction solution against water. Nano^Cys(Bu)^ formed spherical nanoparticles with a narrow distribution, as confirmed by DLS and TEM analyses. The obtained Nano^Cys(Bu)^s were stable against variations in concentration and pH, although they possessed reactive thioester groups in the side chain of the PCys segment. To investigate the antioxidant effect of Nano^Cys(Bu)^, Nano^Cys(Bu)^ was applied to a sepsis model in mice, induced by the intraperitoneal injection of LPS. Interestingly, oral administration of Nano^Cys(Bu)^ prolonged the half-survival time in the sepsis shock model compared with those in the no-treatment and Cys-treatment groups. Therefore, the Nano^Cys(Bu)^ designed in this study shows promising potential for antioxidant therapy, particularly in the context of systemic inflammation, such as sepsis.

## Data Availability

All data are contained in the article.

## Supplementary Materials

The following supporting information can be downloaded at: https://www.mdpi.com/article/10.3390/pharmaceutics15061775/s1, Figure S1: GPC curves and ^1^H NMR spectra of **P2**(SS) and **P3**(SS); Figure S2: GPC curves and ^1^H NMR spectra of **P2**(SH) and **P3**(SH); Figure S3: TEM images of Nano^Cys(Bu)^(**P2**) and Nano^Cys(Bu)^(**P3**); Figure S4: The effect of the polymer concentration on the formation of Nano^Cys(Bu)^; Figure S5: The effect of pH on the formation of Nano^Cys(Bu)^; Table S1: Synthesis and characterization of PEG and PCys-based block copolymers.
