# Supplementary material for: Newly Designed Cysteine-Based Self-Assembling Prodrugs for Sepsis Treatment
Source: Pharmaceutics. 2023 Jun 20;15(6):1775. doi: 10.3390/pharmaceutics15061775 (PMC10302134; doi:10.3390/pharmaceutics15061775)
Supplement: Supplementary file 1 [file pharmaceutics-15-01775-s001.zip › pharmaceutics-2395469-supplementary.pdf]

Supplementary Information

Table of Contents

Supporting Data S3

Figure S1. GPC curves and ^{1}H NMR spectra of **P2**(SS) and **P3**(SS) S3

Figure S2. GPC curves and ^{1}H NMR spectra of **P2**(SH) and **P3**(SH) S4

Figure S3. TEM images of Nano$^{Cys(Bu)}$(**P2**) and Nano$^{Cys(Bu)}$(**P3**) S5

Figure S4. The effect of the polymer concentration on the formation of Nano$^{Cys(Bu)}$ S6

Figure S5. The effect of pH on the formation of Nano$^{Cys(Bu)}$ S7

Table S1. Synthesis and Characterization of PEG and PCys-Based Block Copolymers S8

Supporting Data

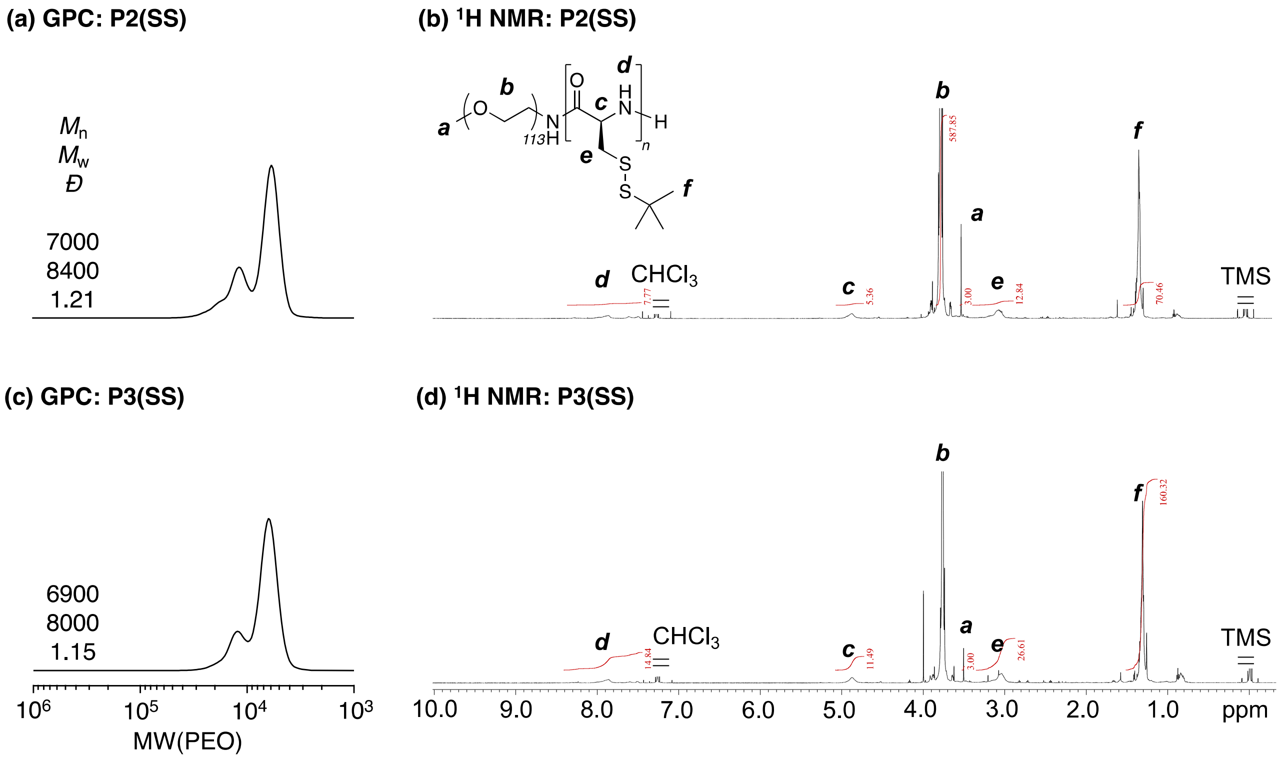

Figure S1. GPC curves and ^{1}H NMR spectra (600 MHz; [polymer] = 5 mg/mL in $CDCl_3$/TFA = 15/1 (v/v)) of PEG-*block*-PCys(S*t*Bu) (a–b, **P2**(SS); c–d, **P3**(SS)).

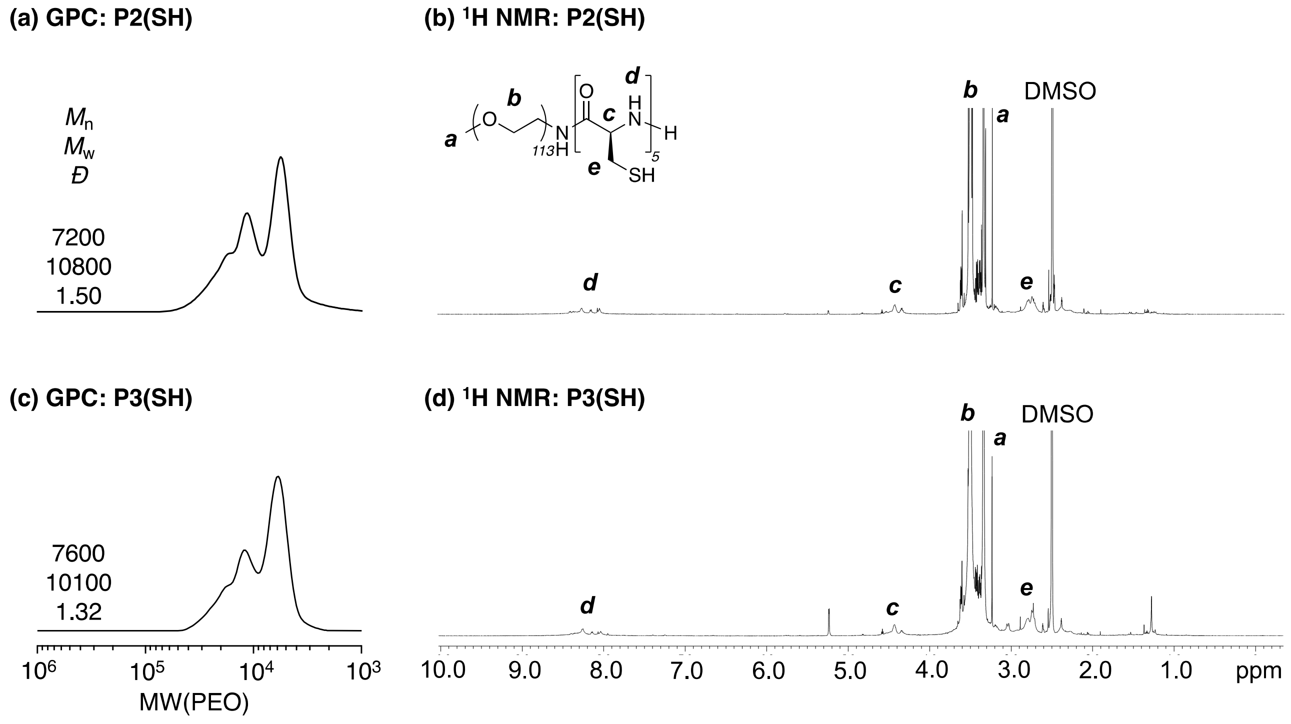

Figure S2. GPC curves and ^{1}H NMR spectra (600 MHz; [polymer] = 5 mg/mL in DMSO-d_6) of PEG-*block*-PCys (a–b, **P2**(SH); c–d, **P3**(SH)).

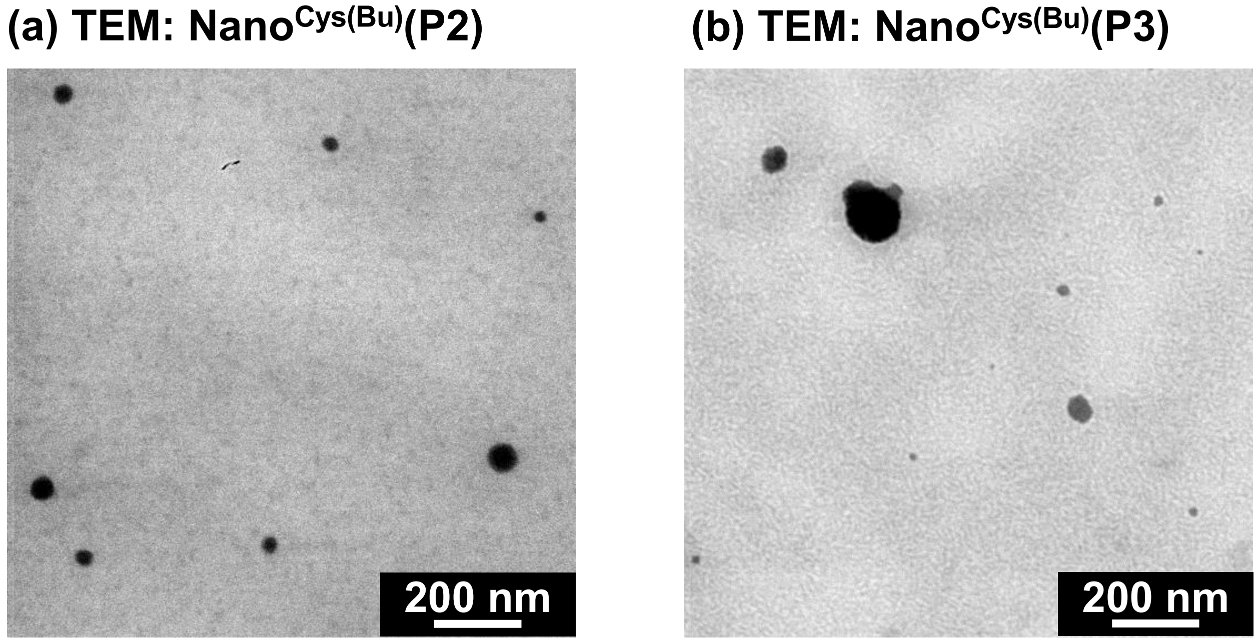

Figure S3. TEM images of (a) Nano$^{Cys(Bu)}$(**P2**) and (b) Nano$^{Cys(Bu)}$(**P3**) cast on the carbon grid from the aqueous solutions and stained by phosphotungstic acid.

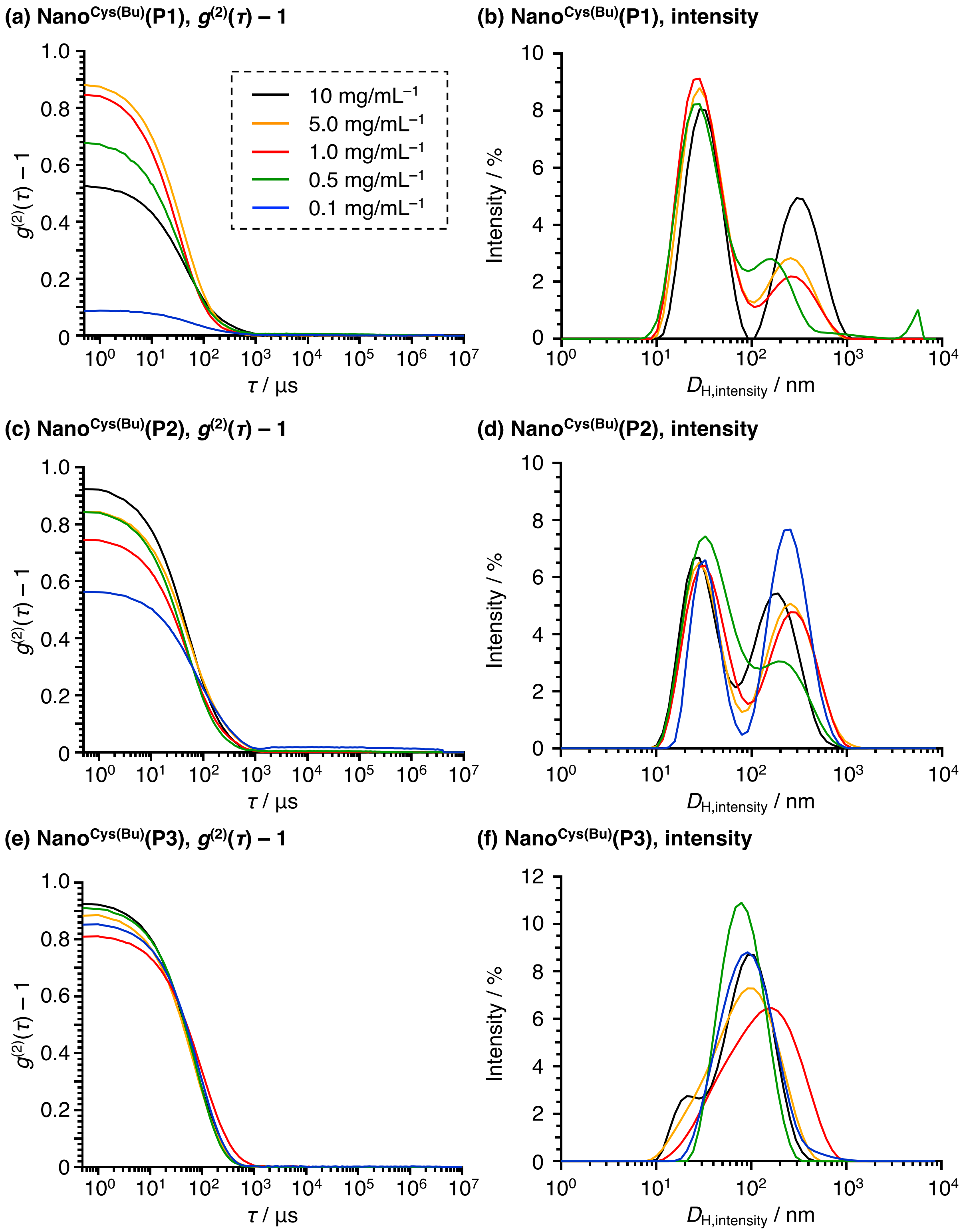

Figure S4. Effect of the polymer concentration on the micelle formation in water (a–b, Nano$^{Cys(Bu)}$(**P1**); c–d, Nano$^{Cys(Bu)}$(**P2**); e–f, Nano$^{Cys(Bu)}$(**P3**)). (a, c, e) The autocorrelation functions ($g^{(2)}(\tau) - 1$) and (b, d, f) the intensity distributions of the hydrodynamic diameter ($D_{H,intensity}$) were characterized by DLS measurements at 37 °C ([polymer] = (blue) 0.1, (green) 0.5, (red) 1.0, (orange) 5.0, and (black) 10 mg/mL).

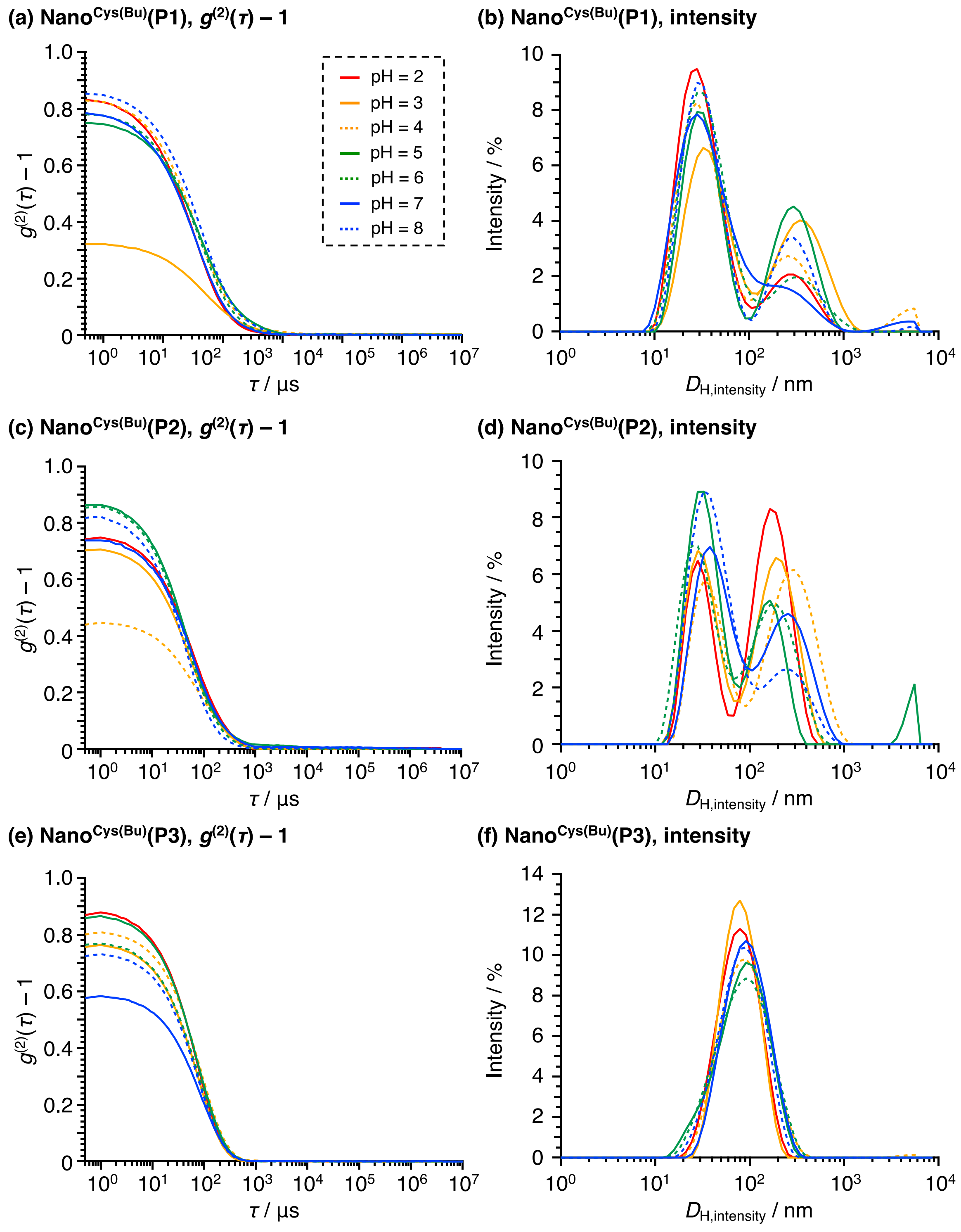

Figure S5. Effect of pH on the micelle size in water (a–b, Nano$^{Cys(Bu)}$(**P1**); c–d, Nano$^{Cys(Bu)}$(**P2**); e–f, Nano$^{Cys(Bu)}$(**P3**)). (a, c, e) The autocorrelation functions ($g^{(2)}(\tau)$ – 1) and (b, d, f) the intensity distributions of the hydrodynamic diameter ($D_{H,intensity}$) were characterized by DLS measurements at 37 °C ([polymer] = 1.0 mg/mL; incubation time = 1 h; incubation temperature = 37 °C; pH = (red solid) 2, (orange solid) 3, (orange dash) 4, (green solid) 5, (green dash) 6, (blue solid) 7, (blue dash) 8).

Table S1. Synthesis and Characterization of PEG and PCys-Based Block Copolymers[a]

Code	Side Chains	NCA / mM	PEG-NH_2 / mM	$\boldsymbol{n}$ [b]	Time / h	Conversion [c] / %[c]	M_n [d] (GPC)	$Đ$ [d] (GPC)	$\boldsymbol{n}_{obsd.}$ [e]	M_n [e] (NMR)	$D_{H,intensity}$ [f] / nm	$D_{H,volume}$ [f] / nm
**P1**	SS	250	42.5	5	96	85	6600	1.24	5	6000	–	–
	SH	–	–		–	–	8200	1.74		5500	–	–
	Bu	–	–		–	–	7600	1.36		5900	34 / 295	20
**P2**	SS	250	21.2	10	95	84	7000	1.21	8	6500	–	–
	SH	–	–		–	–	7200	1.50		5800	–	–
	Bu	–	–		–	–	7500	1.35		6400	37 / 300	26
**P3**	SS	250	10.6	20	96	84	6900	1.15	18	8400	–	–
	SH	–	–		–	–	7600	1.32		6800	–	–
	Bu	–	–		–	–	7300	1.29		8100	166	39

[a] **P1**(SS) – **P3**(SS): [NCA-Cys(S*t*Bu)] / [PEG-NH_2] = 250 / 42.5 (**P1**(SS)), 21.2 (**P2**(SS)), or 10.6 (**P3**(SS)) mM in DMF at 45 °C. [b] Targeted degree of polymerization (DP) at 85% monomer conversion: $\boldsymbol{n}$ = 0.85 × [NCA-Cys(S*t*Bu)]/[PEG-NH_2]. [c] Monomer conversion determined by ^{1}H NMR. [d] Number-average molecular weight (M_n) and distribution ($Đ$) determined by gel permeation chromatography (GPC) in DMF ([LiBr] = 10 mM) with poly(ethylene oxide) (PEO) standards. [e] The observed *DP* ($\boldsymbol{n}_{obsd.}$) was determined using PEG-*block*-PCys(S*t*Bu) by ^{1}H NMR in $CDCl_3$/TFA = 15/1 (v/v) ([polymer] = 5 mg/mL). The number-average molecular weight (M_n(NMR)) was determined by the observed *DP* ($\boldsymbol{n}_{obsd.}$). [f] The intensity- and volume-average hydrodynamic diameter ($D_{H,intensity}$, $D_{H,volume}$) of Nano$^{Cys(Bu)}$s were determined by DLS in water at 37 °C ([polymer] = 1.0 mg/mL).
